# A study on the correlation between family dynamic factors and depression in adolescents

**DOI:** 10.3389/fpsyt.2022.1025168

**Published:** 2023-01-24

**Authors:** Jiali Shi, Yiran Tao, Caiying Yan, Xudong Zhao, Xueqing Wu, Tingting Zhang, Cheng Zhong, Jinhua Sun, Manji Hu

**Affiliations:** ^1^Clinical Research Center for Mental Disorders, Chinese-German Institute of Mental Health, Shanghai Pudong New Area Mental Health Center, School of Medicine, Tongji University, Shanghai, China; ^2^Department of General Medicine, Zhoupu Health Service Center, Shanghai, China; ^3^Department of Psychological Medicine, Children’s Hospital of Fudan University, Shanghai, China

**Keywords:** adolescents, depression, systemic family dynamics, latent class analysis, diagnosis model

## Abstract

**Objectives:**

To evaluate the relationship between systemic family dynamics and adolescent depression.

**Methods:**

An offline survey was distributed to 4,109 students in grades 6–12, with the final analysis including 3,014 students (1,524 boys and 1,490 girls) aged 10–18 years. The questionnaire included the Self-Rating Scale of Systemic Family Dynamics (SSFD), the Self-Rating Depression Scale (SDS), and demographic characteristics.

**Results:**

Family dynamics were negatively correlated with depressive symptoms, with better family dynamics (high scores) associated with lower levels of depression based on the SDS score. After adjusting for sociodemographic characteristics, an ordinal multiclass logistic regression analysis identified family atmosphere (OR = 0.952, 95% CI: 0.948–0.956, *p* < 0.001) as the most important protective family dynamic against depression, followed by individuality (OR = 0.964, 95% CI: 0.960–0.968, *p* < 0.001). Latent class analysis (LCA) created the low family dynamic and high family dynamic groups. There were significant differences in the mean SDS scores between the two groups (45.52 ± 10.57 vs. 53.78 ± 11.88; *p* < 0.001) that persisted after propensity matching. Family atmosphere and individuation had a favorable diagnostic value for depression, with AUCs of 0.778 (95% CI: 0.760–0.796) and 0.710 (95% CI: 0.690–0.730), respectively. The diagnostic models for depression performed well.

**Conclusion:**

Poor family dynamics may be responsible for adolescent depression. A variety of early intervention strategies focused on the family may potentially avoid adolescent depression.

## Introduction

Depression is a serious and growing health issue, and it is one of the leading causes of illness and disability among adolescents (1). The estimated 12-month prevalence of major depression in adolescents worldwide is approximately 13% (2). Adolescent depression is highly prevalent in “low- and middle-income countries,” including China (3). A meta-analysis reported that the prevalence of depressive symptoms among Chinese adolescents was 24.3%, a statistic that increased with grade level (4). In addition to causing considerable suffering and impaired functioning, adolescent depression can be life-threatening when severe. Adolescent depression is closely linked to the risk of suicide, especially in low- and middle-income countries (5).

Adolescents experience more physical, emotional, and social changes than at any other time in their lives (6). Physical symptoms, irritability, and suicidal ideation are common features of adolescent depression. Age-wise adolescent depression exhibits a “steep slope phenomenon.” The incidence of depression before the age of 12–13 is very low, but it then rises rapidly with increasing age (7). In terms of the differences between male and female adolescents, there is almost no difference in the incidence of depression during childhood between boys and girls, but girls are nearly two times as likely to suffer from depression than boys during adolescence (8). Adolescent depression is also associated with other psychiatric disorders. Given its profound, lifelong impact, understanding the factors associated with adolescent depression is very important.

Family is the most basic unit of human development, and children are usually embedded into their family unit and dependent upon their parents for nurturance, support, and assistance (9). Good family dynamics have important and far-reaching implications for the healthy growth of adolescents. Previous studies showed that poor family functioning can lead to an increased risk of depression (10). Specific family based risk factors associated with depression in children include high levels of family conflict (11), low levels of family cohesiveness (12), and maladaptive parent–child communication (13) and problem-solving (14). Parents who exhibit a high degree of aversiveness may, in turn, make their children more vulnerable to depressive disorders (15). Another factor that is strongly associated with depression is the autonomy granted to adolescents as they mature (16). Unfortunately, despite a large body of literature between families and adolescent depression, there has been a relative paucity of research on families as systems.

Systemic family dynamics are an important measure of familial function (17) and reflect interactions between family members (18). The Self-Rating Scale of Systemic Family Dynamics (SSFD) allows for the evaluation of localized (Chinese version) family dynamics, which is based on the Heidelberg theory of family dynamics (19). This allows for an accurate evaluation of the relationship and interactions between family members from a systematic perspective (20). Previous studies evaluated the relationship between family dynamics and mental health problems (Symptom Checklist-90) (21), quality of life (QoL) (22), and stigma in patients with schizophrenia (23). The consistent view is that we should focus on the importance of improving family dynamics. However, there is minimal research on SSFD and adolescent depression. The purpose of this study was to examine the relationship between systemic family dynamics and depression among adolescents and assess the diagnostic value of four family dynamic dimensions for adolescent depression. We administered the standardized questionnaires to a large sample of adolescents enrolled in junior and senior high schools in Shanghai, China.

## Materials and methods

### Participants

A cross-sectional, multicenter survey study was administered from October 2020 to December 2020 in Shanghai, China. The study sample was selected based on convenience sampling from six junior high schools (Grade 6 to Grade 9) and six senior high schools (Grade 10 to Grade 12) in the Yangpu District and Pudong New Area of Shanghai. Both the participants and their guardians agreed to participate in the survey study and signed informed consent prior to completing the surveys. Questionnaires were sent to a total of 4,109 students, with 3,357 returning them (recovery efficiency: 81.7%).

Respondents over the age of 18 years or surveys with greater than 50% missing values (24) were excluded. Variables with greater than 20% missing values were also excluded (25) from our analysis. Responses from a total of 3,014 students aged 10–18 years were analyzed. This study was approved by the Ethics Committee of Shanghai Pudong Mental Health Center in China (PDJWLL2019008).

### Measures

The survey consisted of the following three parts: basic demographic characteristics, the Self-Rating Depression Scale (SDS), and the Self-Rating Scale of Systemic Family Dynamics (SSFD).

Demographic characteristics included age, gender, number of children in the family, parental preference, parental relationship, and monthly family income.

The SDS is a questionnaire that measures depressive symptoms using 20 items. This self-completed questionnaire has good reliability and validity and is widely used (26). Symptoms are rated on a four-point scale. Frequency is converted into an integer between 1 and 4, and the total SDS score is calculated as the sum of responses to all 20 questions. The total SDS score multiplied by 1.25 is rounded to produce the standard score. According to the Chinese norm, SDS scores are interpreted as follows: <53, within the normal range; 53–62, minimal to mild depression; 63–72, moderate to severe depression; ≥73, severe depression. The Cronbach’s α and split-half correlation coefficients of the SDS are 0.73 and 0.84, respectively (27).

The Self-Rating Scale of Systemic Family Dynamics evaluates the perceptions of individuals regarding family dynamics (19) and is suitable for administration to adolescents (28, 29). The SSFD (second edition) has a total of 23 items that are rated on a five-point scale. It includes the following four dimensions: family atmosphere (FA), individuation (IN), system logic (SL), and illness concepts (IC). FA refers to the emotional aspects of communication within the family system (18). A higher score reflects pleasantness and comfort. IN denotes the differentiation between emotions and behaviors. A higher score in this dimension indicates a higher degree of emotional differentiation between family members, less direct parental control over their children, and the permission of children to have their own independent development space. SL reflects the logical characteristics of value judgments among household members. A higher score in this dimension suggests that family members are more inclined to look at family rules and systems with “both…. and…..” logical judgment and a diversified thought process. Finally, IC evaluates the amount of responsibility that members believe they ought to shoulder when managing illness. Higher scores in this dimension suggest that family members tend to think that the psychosomatic state of the whole family is related to their own efforts and psychological factors. This can play a role in their own psychological adjustment. The Cronbach’s α and split-half correlation coefficients for the SSFD were 0.79 and 0.84, respectively (30).

### Statistical analysis

R version 4.1.3 was used for data analysis. The predictive mean matching (PMM) method was used with the “Mice” package to impute missing values (31).

Continuous variables were expressed as the mean ± standard deviations (normal distribution) or median (quartiles) (skewed distribution). Categorical variables were presented as N (%). For comparisons between the two groups, *t*-tests were used for normally distributed continuous variables, the Wilcoxon rank-sum tests were used for non-normally distributed continuous variables, and the chi-square tests were used for categorical variables. A one-way ANOVA was used to compare measurement data between multiple groups (*n* ≥ 3), with the Bonferroni post-test used for multiple comparisons when there were significant differences among them. A multinomial logistic regression analysis was performed to estimate correlations between related risk factors with each SSFD subscale score. Statistical significance was defined as a two-sided *p* < 0.05.

Latent class analysis (LCA) was used to describe the heterogeneity of family dynamic characteristics using the “poLCA” package (32). It is a method that identifies groups of similar subjects based on a set of observed characteristics (33). SSFD items were recoded into binary variables for the LCA, with items with a score of 1 or 2 recorded as 0 and a score of 3 or more as 1. Analysis was performed by increasing the number of classes, starting with a two-class model. Akaike information criterion (AIC), Bayesian information criterion (BIC), and maximum log-likelihood were used to identify the best model. A low value for AIC and BIC or a high value for maximum log-likelihood indicated a better model (34). Entropy was also used to define the best model as it can indicate how precisely the model defines classes (35). Posterior probabilities were used as indicators of classification certainty (36). We stopped fitting the model with an additional class when the posterior probability of the model was <90%. The propensity score matching (PSM) method was then used to adjust for general variables in the secondary analysis to enhance the robustness of our results. The nearest-neighbor matching algorithm was used to select matched pairs of subjects. This analysis was performed using the “MatchIt” package (37).

To explore the role of family dynamics in the risk of depression, the receiver operating characteristic (ROC) curves were created using the “pROC” package (38). We used logistic regression as a variable selection method to select demographic characteristics to establish a basic model (Model 1). The four dimensions of family dynamics were then added to the model. Improvements in model diagnostic performance were evaluated with the area under the curve (AUC), the categorical net reclassification index (NRI), and the relative integrated discrimination improvement (IDI) (39).

## Results

### Demographic characteristics of the subjects

A total of 3,014 adolescents were enrolled in this study, with an average age of 14.13 years (SD = 2.05). The ratio of boys and girls was roughly equal. The proportion of single-child families was high (70.0%), but the ratio of parental preference was very low (only 5.1%). Most of the parents had a close relationship (73.6%). More families have low-to-middle monthly incomes (42.9%). Demographic characteristics are shown in Table 1.

**TABLE 1:** Demographic characteristics of participants (*N* = 3014).

Variables	*N*	%
**Gender**		
Male	1524	50.6
Female	1490	49.4
**Number of children in the family**		
1	2111	70.0
≥2	903	30.0
**Parental preference**		
Yes	155	5.1
No	2859	94.9
**Parental relationship**		
Close	2218	73.6
General	407	13.5
Distant	389	12.9
**Monthly family income**		
Low-income (<5000 RMB)	858	28.5
Low-middle-income (5000–10000 RMB)	1292	42.9
Upper-middle-income (10000–30000 RMB)	515	17.1
High-income (>30000 RMB)	349	11.5

RMB, Chinese Yuan.

### Comparison of standardized scores for each SSFD dimension between different severities of SDS

Standardized scores for each SSFD subscale are shown in Table 2. The incidence of minimal to mild depression was 19.4%, moderate to severe depression was 9.1%, and severe depression was 2.7%. A one-way ANOVA showed that the scores of the four SSFD dimensions were significantly different between different SDS severities. Adolescents with lower FA scores were significantly depressed, with differences between all groups (all *p* < 0.05). IN scores were also significantly different between all groups except for the mild-to-moderate and moderate-to-severe groups (*p* < 0.05). SL and IC were only significantly different between depressed and non-depressed respondents (*p* < 0.05). They were equivalent between different levels of depression (*p* > 0.05).

**TABLE 2:** Comparison of standardized scores of each subscale of SSFD among different severity levels of SDS (mean ± SD).

	No depression (*N* = 2074) ①	Mild depression (*N* = 586) ②	Moderate depression (*N* = 274) ③	Severe depression (*N* = 80) ④
FA	74.89 ± 19.36^a^	56.19 ± 20.41^b^	49.10 ± 22.63^c^	38.87 ± 23.87^d^
IN	70.37 ± 20.58^a^	55.47 ± 21.78^b^	51.51 ± 25.15^bc^	45.83 ± 25.68^c^
SL	64.41 ± 19.11^a^	58.44 ± 18.94^b^	54.73 ± 23.50^b^	53.31 ± 21.97^b^
IC	53.32 ± 23.12^a^	47.46 ± 22.03^b^	47.13 ± 25.18^b^	45.47 ± 24.66^b^

The different normal letters in the same row indicate a significant difference between the two groups at the 0.05 level.

SDS, Self-Rating Depression Scale; SSFD, Self-Rating Scale of Systemic Family Dynamics; FA, family atmosphere; IN, individualization; SL, system logic; IC, illness concepts.

### Family dynamics are associated with depressive symptoms

Table 3 presents the ORs and 95% CIs of depressive symptoms for the family dynamic subscales. Model 1 indicated that FA (OR = 0.950, 95% CI: 0.946–0.954, *p* < 0.001), IN (OR = 0.962, 95% CI: 0.958–0.966, *p* < 0.001), SL (OR = 0.984, 95% CI: 0.980–0.988, *p* < 0.001), and IC (OR = 0.987, 95% CI: 0.984–0.991, *p* < 0.001) scores were significantly associated with depressive symptoms after adjusting for age and gender. Model 2 indicated that FA (OR = 0.952, 95% CI: 0.948–0.956, *p* < 0.001), IN (OR = 0.964, 95% CI: 0.960–0.968, *p* < 0.001), SL (OR = 0.986, 95% CI: 0.982–0.990, *p* < 0.001), and IC (OR = 0.990, 95% CI: 0.986–0.992, *p* < 0.001) were significantly associated with depressive symptoms after adjusting for age, gender, number of children in the family, parental preference, parental relationship, and monthly family income.

**TABLE 3:** Ordinal multiclass logistic regression analysis for the relationship between SSFD subscale and depressive symptoms.

Variables	OR	95% CI	*p*
**Model 1**			
FA	0.950	0.946–0.954	<0.001**
IN	0.962	0.958–0.966	<0.001**
SL	0.984	0.980–0.988	<0.001**
IC	0.987	0.984–0.991	<0.001**
**Model 2**			
FA	0.952	0.948–0.956	<0.001**
IN	0.964	0.960–0.968	<0.001**
SL	0.986	0.982–0.990	<0.001**
IC	0.990	0.986–0.992	<0.001**

Model 1: adjusted for age and gender; Model 2: adjusted for age, gender, number of children in the family, parental preference, parental relationship, and monthly family income.

**p < 0.001.

FA, family atmosphere; IN, individualization; SL, system logic; IC, illness concepts.

### Latent class profiling

**FIGURE 1:**
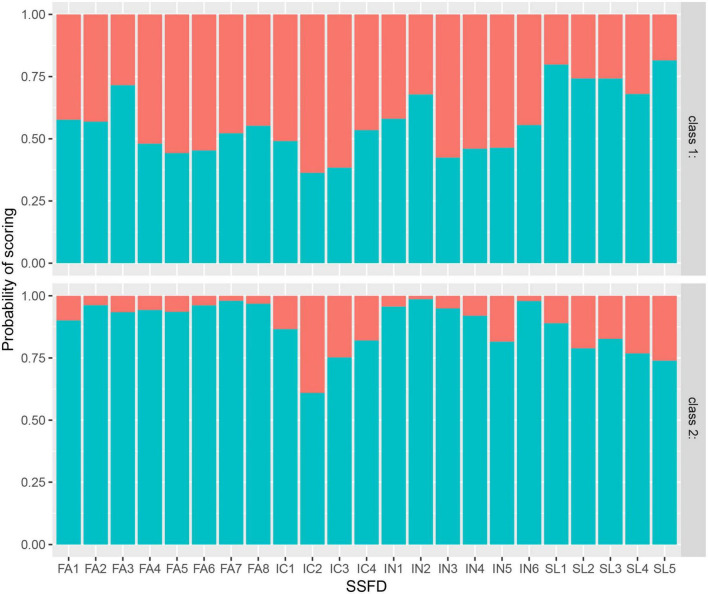
Response probability values of latent classes of family dynamics in participants.

Model fit indices for various models with different latent classes are listed in Table 4. LCA with one to eight classes was performed. AIC and BIC decreased, and maximum log-likelihood increased with classification number, and the two-class model had the highest entropy value (0.885). The posterior probability of each class in the two-class model was >90% (class 1: 97.8%, class 2: 95.9%). Given that some posterior probabilities were less than 90% in the other models, which may reduce the accuracy of the latent class, we chose to use the two-class model. As shown in Figure 1, the response probability values for class 1 were low, suggesting that students had poor family dynamics. This class was labeled the “low family dynamics group.” The response probability values for class 2 were higher, suggesting that students had strong family dynamics. They were thus labeled the “high family dynamics group.”

**TABLE 4:** Fit statistics for latent class models from two to eight classes.

Number of classes	Maximum log-likelihood	AIC	BIC	Entropy
1	−34826	69698	69837	NA
2	−30855	61804	62087	0.885
3	−30347	60837	61265	0.816
4	−29874	59938	60510	0.793
5	−29744	59727	60443	0.783
6	−29313	58912	59773	0.778
7	−29197	58785	59790	0.778
8	−29126	58634	59784	0.756

AIC, Akaike information criterion; BIC, Bayesian information criterion.

### Depressive symptoms differed between the high and low family dynamic groups

As shown in Table 5, the total SDS score was lower in the high family dynamic group than in the low family dynamic group (43.93 ± 10.71 vs. 53.78 ± 11.88, *p* < 0.001). Age, number of children in the family, parental preference, and parental relationship were all significantly different between the two groups (*p* < 0.05). After propensity matching, Table 6 shows that there were no differences between the general characteristics of the high and low family dynamic groups (all *p* > 0.05) and that total SDS scores were still lower in the high family dynamic group than in the low family dynamic group (45.52 ± 10.57 vs. 53.78 ± 11.88, *p* < 0.001).

**TABLE 5:** Comparison of demographic characteristics and total SDS scores among two latent groups.

Variables	High family dynamics group	Low family dynamics group	*p*
	*N* = 2153	*N* = 861	
Age (mean ± SD)	14.29 ± 2.07	13.72 ± 1.95	<0.001**
Gender			0.222
Male	1073 (49.8)	451 (52.4)	
Female	1080 (50.2)	410 (47.6)	
Number of children in the family			0.025*
1	1534 (71.2)	577 (67.0)	
≥2	619 (28.8)	284 (33.0)	
Parental preference			<0.001**
Yes	67 (3.1)	88 (10.2)	
No	2086 (96.9)	773 (89.8)	
Parental relationship			<0.001**
Close	1719 (79.8)	499 (58.0)	
General	228 (10.6)	179(20.8)	
Distant	206 (9.6)	183 (21.2)	
Monthly family income			0.381
Low-income (<5000 RMB)	597 (27.7)	261 (30.3)	
Low-middle-income (5000–10000 RMB)	922 (42.8)	370 (43.0)	
Upper-middle-income (10000–30000 RMB)	377 (17.5)	138 (16.1)	
High-income (>30000 RMB)	257 (12.0)	92 (10.7)	
Total SDS score (mean ± SD)	43.93 ± 10.71	53.78 ± 11.88	<0.001**

*p < 0.05; **p < 0.001.

SDS, Self-Rating Depression Scale; RMB, Chinese Yuan.

**TABLE 6:** Comparison of demographic characteristics and total SDS scores among two latent groups after PSM analysis.

Variables	High family dynamics group	Low family dynamics group	*p*
	*N* = 861	*N* = 861	
Age (mean ± SD)	13.74 ± 1.94	13.72 ± 1.95	0.833
Gender			0.412
Male	433 (50.3)	451 (52.4)	
Female	428 (49.7)	410 (47.6)	
Number of children in the family			0.719
1	585 (67.9)	577 (67.0)	
≥2	276 (32.1)	284 (33.0)	
Parental preference			0.076
Yes	66 (7.7)	88 (10.2)	
No	795 (92.3)	773 (89.8)	
Parental relationship			0.890
Close	504 (58.5)	499 (58.0)	
General	171 (19.9)	179 (20.8)	
Distant	186 (21.6)	183 (21.2)	
Monthly family income			0.602
Low-income (<5000 RMB)	262 (30.4)	261 (30.3)	
Low-middle-income (5000–10000 RMB)	376 (43.7)	370 (43.0)	
Upper-middle-income (10000–30000 RMB)	147 (17.1)	138 (16.0)	
High-income (>30000 RMB)	76 (8.8)	92 (10.7)	
Total SDS scores (mean ± SD)	45.52 ± 10.57	53.78 ± 11.88	<0.001**

**p < 0.001.

SDS, Self-Rating Depression Scale; PSM, propensity score matching.

### Diagnostic value of the four SSFD dimensions for adolescent depression

**FIGURE 2:**
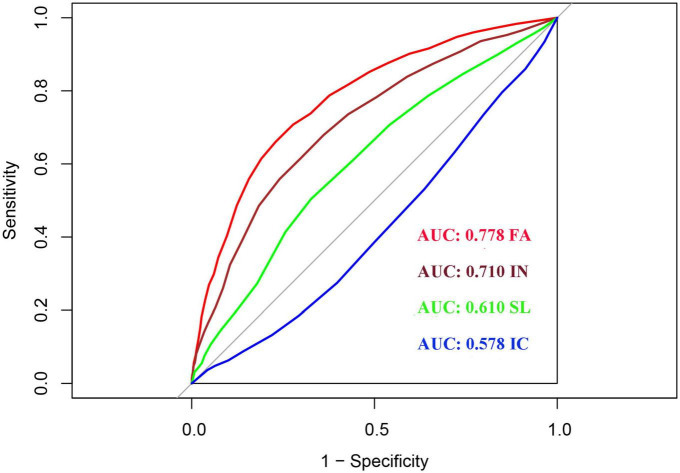
Receiver operating characteristic (ROC) analysis of each dimension of Self-Rating Scale of Systemic Family Dynamics (SSFD).

We used the receiver operating characteristic (ROC) curve analysis to explore the diagnostic value of the four dimensions of SSFD for adolescent depression. As shown in Figure 2, the four SSFD dimensions had different diagnostic values for depression, with an AUC of 0.778 (95% CI: 0.760–0.796) for FA, 0.710 (95% CI: 0.690–0.730) for IN, 0.610 (95% CI: 0.588–0.631) for SL, and 0.578 (95% CI: 0.557–0.600) for IC. FA had the highest AUC. We used demographic characteristics to construct a basic diagnosis model (Model 1) for depression that included age, number of children, parental preference, and parental relationship. As shown in Table 7, the AUC values increased significantly after the addition of FA or IN compared with the basic diagnosis model. The AUC values were not significantly changed by SL or IC. The categorical NRI values of the new models were 0.20 (95% CI: 0.16–0.24), 0.12 (95% CI: 0.08–0.16), 0.03 (95% CI: 0.00–0.06), and 0.03 (95% CI: 0.00–0.06), respectively. The relative IDI values of the new models were 0.15 (95% CI: 0.14–0.16), 0.09 (95% CI: 0.08–0.10), 0.02 (95% CI: 0.01–0.03), and 0.01 (95% CI: 0.00–0.02), respectively.

**TABLE 7:** Comparison of the performance of diagnostic models.

	AUC (95% CI)	Categorical NRI (95% CI)	Relative IDI (95% CI)
Model 1	0.66 (0.64–0.68)	Reference	Reference
Model 1 + FA	0.79 (0.77–0.80)	0.20 (0.16–0.24)	0.15 (0.14–0.16)
Model 1 + IN	0.75 (0.73–0.76)	0.12 (0.08–0.16)	0.09 (0.08–0.10)
Model 1 + SL	0.69 (0.67–0.71)	0.03 (0.00–0.06)	0.02 (0.01–0.03)
Model 1 + IC	0.67 (0.65–0.69)	0.03 (0.00–0.06)	0.01 (0.00–0.02)

Model 1: A basic diagnosis model for depression that includes age, number of children in the family, parental preference, and parental relationship.

AUC, area under the curve; NRI, net reclassification index; IDI, integrated discrimination improvement. FA, family atmosphere; IN, individualization; SL, system logic; IC, illness concepts.

## Discussion

It is important to understand the influencing factors of adolescent depression to enable early diagnosis and treatment. This study explored the relationships between family dynamics and adolescent depression. We evaluated the auxiliary diagnostic value of four family dynamic dimensions for adolescent depression. We found that better family dynamics (high score) were related to a lower risk of depression. The highest protective element of the family dynamic was FA, followed by IN. FA and IN scores demonstrated good diagnostic value for adolescent depression. The more relaxed and happier the family atmosphere of teenagers is, the higher the degree of emotional differentiation between family members, and the fewer control parents have over their children, the less likely teenagers are to suffer from depression.

Our study used LCA to explore the relationship between family dynamics and depression and describe the heterogeneity of family dynamic characteristics. To the best of our knowledge, this is the first study to perform an LCA of the SSFD scale to identify subpopulations influenced by different family dynamics in an adolescent population. The response probability values of all FA and IN items (except IN5) were above 90% in the high family dynamic group but were around 50% in the low family dynamic group. The response probability values of SL and IC items were equivalent between the two classes. This demonstrated the clear heterogeneity of FA and IN among adolescents. Furthermore, there was a significant difference in the total SDS scores of the two groups, with higher total SDS scores in the low family dynamic group. This finding remained the same after PSM for all general characteristics. This interesting discovery further indicates that it is important to create a relaxed and happy family atmosphere and maximize self-differentiation to promote adolescent mental health. Worse family dynamics, especially those evaluated with FA and IN scores, were associated with higher levels of depression. Poor family intimacy or emotional expression can trigger depression in adolescents (40), and families that lack activities and effective communication are prone to developing more psychological problems (41). This result is also similar to those reported in a prior article from our group (42) that reported that poor family functioning was associated with poor FA and that adolescents from dysfunctional families were more likely to be prone to anxiety and depression. Li et al. (17) found that the scores in all four dimensions of the SSFD were significantly improved following combined systemic family therapy in anxious and depressed adolescents with epilepsy. The present study further supports the need to improve family dynamics to help adolescents maintain a good level of mental health during their important period of personality formation. In addition, there is still another essential finding: The mean total SDS score in the low family dynamic group was approximately 53 (the SDS cutoff for depression). This suggests that if the SSFD score of a patient is lower than 40.62, family intervention may be needed in time. Future studies should seek to confirm this conclusion.

Finally, we evaluated the auxiliary diagnostic value of each dimension of systemic family dynamics for adolescent depression. FA and IN scores had good diagnostic values for depression. Adding FA or IN scores to a basic diagnostic model for depression based on demographic characteristics resulted in increased NRI by 20 and 12% and increased IDI by 15 and 9%, respectively. We already knew that NRI and IDI are two alternatives to AUC for measuring the usefulness of a new model (43), and their improvement further demonstrated the important influence of FA and IN in the diagnosis of adolescent depression. Family members should focus on building a harmonious family relationship, respect the personality of their child, and encourage them to solve problems.

This study has several limitations that should be considered when interpreting its findings. First, the cross-sectional design of the study indicates that causal links could not be established. Variables were self-reported, and analysis was limited by missing data. These issues were partially overcome by removing participants with >50% missing data and imputing with a suitable multiple imputation method. Second, depression was assessed at a single time point using the self-reported SDS, which may over interpret adolescent short-term mood changes as depression (44). Further one-on-one interviews with child psychiatrists may permit more acute screening for depression and allow for longitudinal follow-up, producing results more conducive to guiding clinical interventions. Finally, many other factors can affect adolescent depression aside from family dynamics and basic sociodemographics. Disposition, unhealthy lifestyle behaviors (sedentary behaviors), and parental occupation and education level may further impact adolescent depression and alter our findings. These should be considered in future studies.

## Conclusion

A Chinese general population-based study with a large sample size identified a relationship between family dynamics and depression in adolescents. Our findings suggest that additional attention should be paid to family dynamics to avoid mental health consequences. This study may be used as a reference for developing measures for improving and preventing adolescent depression.

## Data availability statement

The raw data supporting the conclusions of this article will be made available by the authors, without undue reservation.

## Ethics statement

The studies involving human participants were reviewed and approved by the Ethics Committee of Shanghai Pudong Mental Health Center in China. Written informed consent to participate in this study was provided by the participants’ legal guardian/next of kin.

## Author contributions

JLS and YT: study conception, manuscript drafting and revision, data reduction, and statistical analysis. CY: sample collection and conceptualization. TZ, CZ, and XW: sample collection. XZ: funding acquisition and supervision. JHS: manuscript revision and supervision. MH: data curation, funding acquisition, investigation, methodology, and project administration. All authors participated in the preparation of the manuscript and approved its content.

## References

[1] Gore F Bloem P Patton G Ferguson J Joseph V Coffey C Global burden of disease in young people aged 10-24 years: a systematic analysis. *Lancet.* (2011) 377:2093–102. 10.1016/s0140-6736(11)60512-6 21652063

[2] McCance-Katz E. The substance abuse and mental health services administration (SAMHSA): new directions. *Psychiatr Serv.* (2018) 69:1046–8. 10.1176/appi.ps.201800281 30099944

[3] Liu S Chen S Stubbs B Yu Q Griffiths M Jiao C Association between active school travel and depressive symptoms among 51,702 adolescents in 26 low-and middle-income countries. *Int J Ment Health Promot.* (2020) 23:141–53.

[4] Tang X Tang S Ren Z Wong D. Prevalence of depressive symptoms among adolescents in secondary school in mainland China: a systematic review and meta-analysis. *J Affect Disord.* (2019) 245:498–507. 30439677 10.1016/j.jad.2018.11.043

[5] Chen S Guo T Yu Q Stubbs B Clark C Zhang Z Active school travel is associated with fewer suicide attempts among adolescents from low-and middle-income countries. *Int J Clin Health Psychol.* (2021) 21:100202. 10.1016/j.ijchp.2020.11.001 33363585 PMC7753036

[6] Santrock JW *Adolescence: an introduction.* Wm C Brown Publishers (1987).

[7] Mojtabai R Olfson M Han B. National trends in the prevalence and treatment of depression in adolescents and young adults. *Pediatrics.* (2016) 138:e20161878. 10.1542/peds.2016-1878 27940701 PMC5127071

[8] Avenevoli S Swendsen J He J Burstein M Merikangas K. Major depression in the national comorbidity survey-adolescent supplement: prevalence, correlates, and treatment. *J Am Acad Child Adolesc Psychiatry.* (2015) 54:37–44.e2. 10.1016/j.jaac.2014.10.010 25524788 PMC4408277

[9] Hammen C Rudolph K Weisz J Rao U Burge D. The context of depression in clinic-referred youth: neglected areas in treatment. *J Am Acad Child Adolesc Psychiatry.* (1999) 38:64–71. 10.1097/00004583-199901000-00021 9893418

[10] Yl A Xs B Mr C Ht C Hc D Tong W Depressive symptoms between parent and adolescent survivors: a longitudinal actor-partner interdependence model. *J Affect Disord.* (2020) 265:139–45. 10.1016/j.jad.2020.01.038 32090735

[11] Barratt S Marital conflict and children: an emotional security perspective. *Child Adolesc Ment Health.* (2011) 16:173.

[12] Rao U Chen L. Characteristics, correlates, and outcomes of childhood and adolescent depressive disorders. *Dialog Clin Neurosci.* (2009) 11:45–62. 10.31887/DCNS.2009.11.1/urao PMC2766280 19432387

[13] Asarnow J Goldstein M Tompson M Guthrie D. One-year outcomes of depressive disorders in child psychiatric in-patients: evaluation of the prognostic power of a brief measure of expressed emotion. *J Child Psychol Psychiatry Allied Discip.* (2010) 34:129–37. 10.1111/j.1469-7610.1993.tb00975.x 8444988

[14] Hammen C. Stress generation in depression: reflections on origins, research, and future directions. *J Clin Psychol.* (2010) 62:1065–82. 10.1002/jclp.20293 16810666

[15] Yap M Allen N Sheeber L. Using an emotion regulation framework to understand the role of temperament and family processes in risk for adolescent depressive disorders. *Clin Child Fam Psychol Rev.* (2007) 10:180–96. 10.1007/s10567-006-0014-0 17265137

[16] Restifo K Bögels S. Family processes in the development of youth depression: translating the evidence to treatment. *Clin Psychol Rev.* (2009) 29:294–316. 10.1016/j.cpr.2009.02.005 19356833

[17] Li J Wang X Meng H Zeng K Quan F Liu F. Systemic family therapy of comorbidity of anxiety and depression with epilepsy in adolescents. *Psychiatry Investig.* (2016) 13:305. 10.4306/pi.2016.13.3.305 27247596 PMC4878964

[18] Yang Y Liang Y Zhang Y Wang Y Shang L. Revision, reliability and validity evaluation of self-rating scale of systemic family dynamics-students testing version. *Chin J Public Health.* (2016) 32:167–70.

[19] Kang CZ Xu X Yang K Yang J. The questionnaire of systemic family dynamics: development, reliability and validity. *Chin Ment Health J.* (2001) 015:92–5.

[20] Yang J. The self-rating inventory of systematic family dynamics: development, reliability and validity. *Chin J Clin Psychol.* (2002) 10:263–5.

[21] Yang Z Cui Y Yang Y Wang Y Zhang H Liang Y The relationship between mental health problems and systemic family dynamics among high school and university students in shaanxi province, china. *Int J Public Health.* (2021) 66:1603988. 10.3389/ijph.2021.1603988 34552461 PMC8450291

[22] Yang Z Shang Y Liang Y Zhang H Yang Y Wang Y The quality of life and its relationship with systemic family dynamics and mental health in senior high school students from shaanxi, china. *Front Public Health.* (2022) 10:833561. 10.3389/fpubh.2022.833561 35433624 PMC9008304

[23] Wang L Chen Y Hu C Qin H. Influence of family dynamics on stigma experienced by patients with schizophrenia: mediating effect of quality of life. *Front Psychiatry.* (2021) 12:645075. 10.3389/fpsyt.2021.645075 34483978 PMC8415875

[24] Harel O Pellowski J Kalichman S. Are we missing the importance of missing values in HIV prevention randomized clinical trials? Review and recommendations. *AIDS Behav.* (2012) 16:1382–93. 10.1007/s10461-011-0125-6 22223301 PMC3358416

[25] Lee B Vittinghoff E Dodge J Cullaro G Terrault N. National trends and long-term outcomes of liver transplant for alcohol-associated liver disease in the United States. *JAMA Internal Med.* (2019) 179:340–8. 10.1001/jamainternmed.2018.6536 30667468 PMC6439700

[26] Bae Y Kim S. Low dietary calcium is associated with self-rated depression in middle-aged Korean women. *Nutr Res Pract.* (2012) 6:527–33. 10.4162/nrp.2012.6.6.527 23346303 PMC3542443

[27] Wang X Wang X Ma H. *Handbook of mental health assessment scale.* Beijing: China Mental Health Journal Press (1999).

[28] Yang J Kang C Zhao X. The self-rating inventory of systematic family dynamics: development, reliability and validity. *Chin J Clin Psychol.* (2002) 10:263–5.

[29] Yang YL Zhang Y Wang Y Shang L. Revision, reliability and validity evaluation of self-rating scale of systemic family dynamics-students testing version. *Chin J Public Health.* (2016) 32:4.

[30] Yu L Zhao XD Zeng WN Tan JF Yu YL Zeng JY Reliability and validity analysis of the revised version of the systematic family dynamics self-assessment scale in a test of guangdong students. *China Health Stat.* (2014) 31:979–81.

[31] Van Buuren S Groothuis-Oudshoorn K. Mice: multivariate imputation by chained equations. *J Statist Softw.* (2017) 45:1–67.

[32] Linzer D Lewis J. poLCA: an R package for polytomous variable latent class analysis. *J Statist Softw.* (2011) 42:1–29.

[33] Kim M Wall M Li G. Applying latent class analysis to risk stratification for perioperative mortality in patients undergoing intraabdominal general surgery. *Anesth Analg.* (2016) 123:193–205. 10.1213/ane.0000000000001279 27111648

[34] Weller B Bowen N Faubert S. Latent class analysis: a guide to best practice. *J Black Psychol.* (2020) 46:287–311. 10.1186/s12877-016-0323-1 27492449 PMC4974723

[35] Nylund K Asparouhov T Muthén B. Deciding on the number of classes in latent class analysis and growth mixture modeling: a monte carlo simulation study: structural equation modeling: a multidisciplinary. *Struct Equ Model.* (2007) 14:535–69.

[36] Spaan P Blokland A De Blander R Robert L Maes E Blom M Differentiating individuals convicted of sexual offenses: a two-country latent class analysis. *Sex Abuse.* (2020) 32:423–51. 10.1177/1079063219893370 31845624

[37] Schullian P Putzer D Silva M Laimer G Kolbitsch C Bale R. Stereotactic radiofrequency ablation of liver tumors in octogenarians. *Front Oncol.* (2019) 9:929. 10.3389/fonc.2019.00929 31608232 PMC6761359

[38] Robin X Turck N Hainard A Tiberti N Lisacek F Sanchez J pROC: an open-source package for R and S+ to analyze and compare ROC curves. *BMC Bioinform.* (2011) 12:77. 10.1186/1471-2105-12-77 21414208 PMC3068975

[39] Zhang L Zhang F Xu F Wang Z Ren Y Han D Construction and evaluation of a sepsis risk prediction model for urinary tract infection. *Front Med.* (2021) 8:671184. 10.3389/fmed.2021.671184 34095176 PMC8175780

[40] Kouros C Garber J. Trajectories of individual depressive symptoms in adolescents: gender and family relationships as predictors. *Dev Psychol.* (2014) 50:2633–43. 10.1037/a0038190 25329553 PMC4591045

[41] King K Vidourek R Merianos A. Authoritarian parenting and youth depression: results from a national study. *J Prev Interv Commun.* (2016) 44:130–9. 10.1080/10852352.2016.1132870 26939843

[42] Hu M Xu L Zhu W Zhang T Wang Q Ai Z The influence of childhood trauma and family functioning on internet addiction in adolescents: a chain-mediated model analysis. *Int J Environ Res Public Health.* (2022) 19:13639. 10.3390/ijerph192013639 36294219 PMC9602662

[43] Pencina M D’Agostino R Sr Steyerberg E. Extensions of net reclassification improvement calculations to measure usefulness of new biomarkers. *Stat Med.* (2011) 30:11–21. 10.1002/sim.4085 21204120 PMC3341973

[44] Gariépy G Honkaniemi H Quesnel-Vallée A. Social support and protection from depression: systematic review of current findings in Western countries. *Br J Psychiatry.* (2016) 209:284–93. 10.1192/bjp.bp.115.169094 27445355

